# Awareness among French healthcare workers of the transmission of multidrug resistant organisms: a large cross-sectional survey

**DOI:** 10.1186/s13756-019-0625-0

**Published:** 2019-11-12

**Authors:** L. Vaillant, G. Birgand, M. Esposito-Farese, P. Astagneau, C. Pulcini, J. Robert, J. R. Zahar, E. Sales-Wuillemin, F. Tubach, J. C. Lucet, the PerceptR Study group, Aveline Isabelle, Bracco Christelle, Lacombe Manuelle, Colin Yolande, Poulingue Géraldine, Lesourd Fabien, Rogues Anne-Marie, Magne Béatrice, Mouzaoui Marc, Daniel Petrelli, Picot Franck, Canivet Anne, Stoeckel Vincent, Marchal Lydia, Boris Alexandre, Tiv Michel, Piriou Gilles, Lallart Dominique-Louis, Lecoq Marianne, Pina Patrick, Barege Patrice, Josso Christophe, Herbin Claire, Degallaix Dominique, Doublier Laetitia, Constantin Nicole, Ludvig-Serge Aho-Glélé, Parer Sylvie, Minchella Amandine, Romand Karine, Bentchikou Hicham, Petitfrère Manuel, Lepelletier Didier, Vaillé Jean-louis, Houdou Sylvie, Rahal Gisèle, Miquel Chantal, Jaudinot Christel, Dehaese Olivier, Thouvenin Dominique, Pascal Eliane, Marini Hélène, Merle Véronique, Leroux Elisabeth, Laurent Oleessya, Cavarec-Cuoq Marie-Claude, Carrière Isabelle, Millet Elisabeth, Fontaine Xavier, Markiewicz Amélie, Germain Yves, Coppens Magali, Jean Sébastien Trescher, Duperrier Valérie, Paba Odile, Fanck Marie-Noëlle, Jezequel Jocelyn, Velardo Danielle, Gachot Bertrand, Lestra Bénédicte

**Affiliations:** 1 AP-HP, Bichat-Claude Bernard Hospital, Infection Control Unit, 48 rue Henri Huchard, F-75018 Paris, France; 2 Department of Medicine, NIHR, Imperial College London, Health Protection Research Unit in Antimicrobial Resistance and Healthcare Associated Infection Imperial College London, South Kensington Campus, London, SW7 2AZ UK; 3 AP-HP, Bichat-Claude Bernard Hospital, Unité de Recherche Clinique Paris Nord Val de Seine and CIC-EC 1425, 48 rue Henri Huchard, F-75018 Paris, France; 4 Medecine Sorbonne University, AP-HP, Regional centre for Prevention of Healthcare-associated infections, 8 rue Maria Helena Vieira da Silva, 75014 Paris, France; 5 EA 4360 APEMAC, CHRU de Nancy, University of Lorraine, Infectious and Tropical Diseases Unit, 34 Cours Léopold, 54000 Nancy, France; 6 Sorbonne University, U1135, Team E13, CR7 INSERM, AP-HP, Pitié-Salpêtrière Hospital, Bactériologie-Hygiène, 47-83 Boulevard de l’Hôpital, 75013 Paris, France; 7 AP-HP, Avicenne Hospital, Infection Control Unit, 125 Rue de Stalingrad, 93000 Bobigny, France; 8 University of Paris, INSERM, IAME, UMR 1137, Paris, France; 9 University of Bourgogne Franche-Comté SPMS, Dijon, France; 10 INSERM, UMR 1123, AP-HP, Pitié-Salpêtrière Hospital, Centre de Pharmacoépidémiologie (Cephepi), 75013 Paris, France

**Keywords:** Healthcare workers, Health personnel/classification/education, Drug resistance, bacterial, Cross-infection/*prevention & control, Cross-sectional studies, Hand disinfection, Health knowledge, attitudes, practice, Surveys and questionnaires, France

## Abstract

**Background:**

Much effort has been made over the last two decades to educate and train healthcare professionals working on antimicrobial resistance in French hospitals. However, little has been done in France to assess perceptions, attitudes and knowledge regarding multidrug resistant organisms (MDROs) and, more globally, these have never been evaluated in a large-scale population of medical and non-medical healthcare workers (HCWs). Our aim was to explore awareness among HCWs by evaluating their knowledge of MDROs and the associated control measures, by comparing perceptions between professional categories and by studying the impact of training and health beliefs.

**Methods:**

A multicentre cross-sectional study was conducted in 58 randomly selected French healthcare facilities with questionnaires including professional and demographic characteristics, and knowledge and perception of MDRO transmission and control. A knowledge score was calculated and used in a logistic regression analysis to identify factors associated with higher knowledge of MDROs, and the association between knowledge and perception.

**Results:**

Between June 2014 and March 2016, 8716/11,753 (participation rate, 74%) questionnaires were completed. The mean knowledge score was 4.7/8 (SD: 1.3) and 3.6/8 (SD: 1.4) in medical and non-medical HCWs, respectively. Five variables were positively associated with higher knowledge: working in a university hospital (adjusted odds ratio, 1.41, 95% CI 1.16–1.70); age classes 26–35 years (1.43, 1.23–1.6) and 36–45 years (1.19, 1.01–1.40); medical professional status (3.7, 3.09–4.44), working in an intensive care unit (1.28, 1.06–1.55), and having been trained on control of antimicrobial resistance (1.31, 1.16–1.48). After adjustment for these variables, greater knowledge was significantly associated with four cognitive factors: perceived susceptibility, attitude toward hand hygiene, self-efficacy, and motivation.

**Conclusions:**

We found a low level of MDRO awareness and knowledge of associated control measures among French HCWs. Training on hand hygiene and measures to control MDRO spread may be helpful in shaping beliefs and perceptions on MDRO control among other possible associated factors. Messages should be tailored to professional status and their perception. Other approaches should be designed, with more effective methods of training and cognitive interventions.

**Trial registration:**

Clinical Trials.gov NCT02265471. Registered 16 October 2014 - Retrospectively registered.

## Background

Antimicrobial resistance (AMR) is a growing problem worldwide. Multidrug-resistant organisms (MDROs) are challenging healthcare workers (HCWs) in their daily practice and there is an urgent need for improved infection prevention and control (IPC) practices and antimicrobial stewardship. Many guidelines and training materials have been issued for the control of MDRO transmission. Successful interventions have served as a framework for the implementation of further control programmes [1, 2]. However, recommendations alone are not enough to improve compliance with best practices. As demonstrated in the context of hand hygiene, guidelines must be associated with an implementation process considering contextual and behavioural determinants [3]. A strong association between knowledge, perceptions, and ultimately actions has been suggested in previous research on AMR [4, 5]. Some studies found an impact of knowledge, attitudes, and personal perceptions, including perceived benefits and barriers, on the behaviours and practices of HCWs in IPC [6–9].

Surveys on the knowledge and perception of AMR have primarily focused on antibiotic prescription, excluding infection control measures [10]. More recently, several studies have jointly assessed knowledge, attitudes, and practices regarding both MDROs and transmission precautions [11–13]. The findings indicated that few physicians were concerned with their own infection control practices, though they were aware of the threat of AMR. Most of them targeted junior doctors or medical students [14].

Our aim was to explore awareness among HCWs by evaluating their general knowledge on MDROs and associated control measures, comparing perceptions between professional categories, and studying the impact of training and beliefs. Hence, a questionnaire-based survey was developed to identify the association between knowledge, perceptions, and attitudes towards MDROs and control measures (gloves, hand hygiene).

## Methods

### Hospitals and participants

The study was conducted in 58 randomly selected French healthcare facilities (HCFs). Among them, nine were university hospitals or referral centres for cancer (UHs), 10 non-university public hospitals (NUPHs), 10 private HCFs, and 29 in a group mixing local hospitals (*n* = 10), nursing homes (*n* = 10), rehabilitation and long-term care facilities (LTCFs, *n* = 9). Random sampling was used to select participating HCFs, stratified into five geographical areas corresponding to the French interregional coordinating centres for infection prevention and control (CCLIN). This sample represented 2.0% of the total number of French HCFs (58/2931).

Each initial randomised HCF (*n* = 60) was contacted through the local infection prevention and control (IPC) team. A total of 46 HCFs agreed to participate during the first round of randomisation. When an HCF declined to participate (*n* = 21), another HCF was randomly selected following the same scheme of stratification sampling. Twelve other HCFs agreed to participate across three other rounds of sampling. The number of clinical units participating in the survey was correlated with the total number of beds in the facility, from 15 to 50% of clinical units randomly selected in large HCFs, to 100% in small HCFs. Adult and paediatric clinical units were eligible, including intensive care (ICU), medical and surgical units, rehabilitation and long-term care, emergency, outpatient, and radiology units. Eligible HCWs included physicians (senior, junior, and medical students) and non-medical professionals i.e. nurses, nurse aides, nursing students, head nurses, hospital service agents (including cleaning staff and domestic services) and medical-technical agents (including technical staff members, i.e. dieticians, X-ray technicians, physiotherapists, psychologists, …) present during the day and night shifts. HCWs in laboratories, housekeeping personnel, and administrative personnel were excluded.

### Study design

This cross-sectional study was performed from June 2014 to March 2016. Interviewers included members of the study team and members of the local IPC team. The day of the survey, interviewers went through the different included units several times a day (including the night) to ask participants to complete anonymously a self-administered questionnaire requiring 10–15 min to fill. The total number of HCWs present on site the day of the survey was used to compute the participation rate. This information was provided by the local human resources services.

### Questionnaire

The questionnaire was structured in three different parts: (i) professional characteristics including gender, age, professional status, job tenure, working unit, main activity of the unit, working shift, and previous training sessions during the last 3 years about hand hygiene and contact precautions; (ii) assessment of knowledge on the transmission and control of MDROs (Additional file 1: Table S1) including hand hygiene (three questions), glove use (two questions), and epidemiology of MDROs (three questions); (iii) and the perception of AMR included (Additional file 1: Table S2) the perceived threat of MDROs (three questions), individual cognitive factors for hand hygiene compliance (eight questions), based on the theory of health belief model [15–17]. This model enabled the assessment of the following criteria: perceived susceptibility, perceived knowledge, intention to adhere (perceived practice), attitudes toward hand hygiene, perceived behavioural norm, perceived subjective norm, self-efficacy, and motivation (Additional file 1: Table S3) regarding one specific topic. Items related to beliefs and perception were coded on a 7-point Likert scale, ranging from 1, “strongly disagree” to 7, “strongly agree” with the statement of the item. The questions were selected by the steering group which included experts in infectious diseases, public health, infection control and statistics. Questions on infection control (hand hygiene and gloves) and the epidemiology of AMR were selected according to current national guidelines. The questionnaire was first tested among individuals from various professional backgrounds, and some questions were revised slightly according to their comments.

### Statistical analysis

Continuous variables were expressed as mean and standard deviation (SD) or median and interquartile range (Q1: 25th percentile; Q3: 75th percentile), and categorical variables as frequency (percentage). Comparisons between two groups were made using the Chi2 test or Student’s t test or their corresponding non-parametric versions, Fisher’s test or the Wilcoxon rank sum test, as appropriate. Comparisons between more than two groups were made using the Hochberg method for multiple comparisons in order to adjust for the alpha level. The principal endpoint was the knowledge score (KS) defined by the sum of correct answers out of eight questions (Additional file 1: Table S1). The KS was compared among HCF categories, age classes, professional statuses, working units, and other professional characteristics, using the Kruskal–Wallis test.

The KS was then categorised in two classes by its median value, KS lower than four or KS equal to or greater than four. Multivariate logistic regression models were used to assess the association between professional characteristics and KS.

For multivariate analyses, variable selection was done in order to select the best subset of predictors of knowledge. Initial selection was determined by the clinical value of predictors. Then, final selection of explanatory variables in the multivariate analysis was done using stepwise methods based on the AIC (Akaike Information Criterion). All questions about perception and beliefs on the 7-point Likert scale were dichotomised: no agreement with the proposition (“Strongly disagree”, “Disagree”, “Somewhat disagree”, “Neither agree nor disagree”, and “Somewhat agree”) and agreement with the proposition (“Agree” and “Strongly agree”). The latter denoted strong positive agreement with the proposition. All other quotations scores (from 1 to 5) were considered negative according to previous studies in the field of infection control [17]. Significant associations between perception and KS in the univariable analysis were then adjusted for significant variables in the first multivariable model. Reference groups for multivariate analysis were selected from an epidemiological perspective. R software (v3.14) was used.

## Results

### Healthcare facilities and participants

Among the 58 participating HCFs, a total of 8716 HCWs completed the questionnaire. The overall participation rate was 74% (8716/11,753), ranging from 35 to 100% across individual HCFs, with participations of 55% (1291/2335) for the medical healthcare workers (MWs) and 79% (7425/9418) for the non-medical healthcare workers (NMWs). The characteristics of the population are presented in Table 1. Most participants were female (7103/8716; 83%), representing 50% (63/291) and 88% (6469/7425) of MWs and NMWs, respectively. The median age was 33 (Q1; Q3, 27; 47) years old and 37 (28; 48) years old in MWs and NMWs, respectively. Overall, 5753 (68%) and 2787 (34%) HCWs declared having been trained on hand hygiene and control of AMR over the 3 years prior to the survey, respectively.

**Table 1:** Population characteristics

	Total (58 HCFs)	University hospitals/Cancer centres (*n* = 9)	Non-university hospitals (*n* = 10)	Small, rehabilitation, nursing hospitals (*n* = 29)	Private clinics (*n* = 10)
Participants n (%)					
- Total	8716 (100)	4015 (46)	2187 (25)	1885 (22)	629 (7)
- Medical	1291 (15)	818 (20)	285 (13)	99 (5)	89 (14)
- Non-medical	7425 (85)	3197 (80)	1902 (87)	1786 (95)	540 (86)
Male gender n (%)					
- Total	1499 (17)	771 (19)	371 (17)	219 (12)	138 (22)
- Medical	637 (50)	375 (47)	143 (51)	54 (56)	65 (75)
- Non-medical	862 (12)	396 (12)	228 (12)	165 (9)	73 (14)
Age median (Q1;Q3)					
- Total	37 (28; 48)	34 (27; 46)	39 (30; 47)	40 (29; 50)	39 (30; 51)
- Medical	33 (27; 47)	29 (25; 38)	41 (30; 50)	52 (42; 60)	54 (41; 61)
- Non-medical	37 (28; 48)	35 (28; 47)	39 (30; 47)	39 (29; 49)	37 (29; 50)
Professional status n (%)					
- Senior physician	787 (9)	395 (10)	213 (10)	93 (5)	86 (14)
- Junior doctor	332 (4)	271 (7)	57 (3)	2 (0)	2 (0)
- Medical student	165 (2)	149 (4)	12 (1)	3 (0)	1 (0)
- Nurse	2842 (34)	1468 (37)	751 (35)	352 (20)	271 (44)
- Nurse aide	2231 (26)	800 (20)	674 (31)	641 (36)	116 (19)
- Hospital service agent	707 (8)	208 (5)	123 (6)	322 (19)	54 (9)
- Medical-technical agent	506 (6)	245 (6)	93 (4)	157 (9)	11 (2)
- Non-medical student	407 (5)	178 (5)	105 (5)	93 (5)	31 (5)
- Other	487 (6)	233 (6)	111 (5)	103 (6)	40 (7)
Job tenure (median (Q1; Q3))					
- Total	10 (4; 20)	8 (3; 20)	11 (5; 20)	10 (4; 20)	12 (5; 26)
- Medical	6 (2; 19)	4 (2; 11)	12 (3; 20)	24 (10; 30)	26 (13; 32)
- Non-medical	10 (4; 20)	10 (4; 20)	11 (5; 20)	10 (4; 18)	10 (4; 22)
Working unit n (%)					
- Medicine	2742 (33)	1720 (45)	790 (38)	104 (6)	128 (21)
- Intensive care unit	776 (9)	535 (14)	175 (8)	0 (0)	66 (11)
- Emergency	395 (5)	245 (6)	139 (7)	2 (0)	9 (2)
- Rehabilitation, long-term care	2491 (30)	227 (6)	522 (25)	1687 (92)	55 (9)
- Surgery	1610 (19)	923 (24)	366 (17)	0 (0)	321 (53)
- Gynaecology-Obstetrics	123 (1)	66 (2)	47 (2)	1 (0)	9 (1)
- Psychiatry	96 (1)	58 (1)	23 (1)	15 (1)	0 (0)
- Other	158 (2)	84 (2)	45 (2)	14 (1)	15 (2)

Q1: 25th percentile; Q3: 75th percentile

### Awareness and associated factors

The mean KS on AMR and control measures was 4.7/8 among MWs and 3.6/8 among NMWs (*P* < 0.0001) (Table 2). They both differed between the type of HCFs (*p* < 0.001), with a medical KS significantly higher in UHs, and a non-medical KS significantly lower in the LTCF group.

**Table 2:** Knowledge score (KS) regarding antimicrobial resistance and infection control measures

	N (%)	Knowledge score (mean (SD))	*p*-value (global)
Type of healthcare facility			
- University hospitals / cancer centres	4015 (46)	4.0 (1.4)	*p* < 0.001
- Non-university hospitals	2187 (25)	3.7 (1.4)	
- Small, rehabilitation, nursing hospitals	1885 (22)	3.4 (1.5)	
- Private clinics	629 (7)	3.8 (1.4)	
Professional status			
- Medical (total)	1284 (15)	4.7 (1.3)	*p* < 0.0001*
- Senior physician	787 (61)	4.6 (1.4)	
- Junior doctor	332 (26)	4.9 (1.2)	
- Medical student	165 (13)	4.6 (1.3)	
- Non-medical (total)	7180 (85)	3.6 (1.4)	
- Nurse	2842 (40)	4.1 (1.3)	
- Nurse aide	2231 (31)	3.3 (1.3)	
- Hospital service agent	707 (10)	2.6 (1.3)	
- Medical-technical agent	506 (7)	3.6 (1.4)	
- Non-medical student	407 (6)	3.9 (1.3)	
- Other	487 (7)	4.0 (1.5)	
Working unit			
- Medicine	2742 (32)	3.9 (1.4)	*p* < 0.0001
- Surgery	1610 (18)	3.8 (1.4)	
- Intensive care unit	776 (9)	4.2 (1.5)	
- Rehabilitation, long-term care	2491 (29)	3.5 (1.5)	
- Emergency	395 (5)	4;1 (1.4)	
- Gynaecology-Obstetrics	123 (1)	3.6 (1.4)	
- Psychiatry	96 (1)	3.5 (1.5)	
- Other	158 (2)	3.5 (1.6)	
Previous training for hand hygiene (last 3 years)			
- Yes	5753 (68)	3.8 (1.5)	*p* < 0.0001
- No	2769 (32)	3.7 (1.4)	
Previous training in control of AMR (last 3 years)			
- Yes	2787 (34)	4.0 (1.5)	*p* < 0.0001
- No	5413 (66)	3.7 (1.4)	

*p value medical vs. non-medical

Most respondents wrongly thought that hand hygiene was more important after than before contact with a patient (58% MWs, 52% NMWs); alcohol-based hand rub (AHR) was correctly considered more effective than antiseptic or plain soap (76% MWs, 50% NMWs) (Additional file 1: Table S1). A large proportion, (> 90%) believed that gloves were indicated for contact precautions. Standard precautions (hand hygiene after contact with the patient’s environment and no glove wearing for contact with the patient’s intact skin) were correctly known (higher than 80% in both MWs and NMWs).

Knowledge on the MDRO epidemiology was greater among MWs; 85% of MWs and 67% of NMWs considered that methicillin-resistant *Staphylococcus aureus* (MRSA) was mainly hand-transmitted. A large proportion of respondents thought that rates of both MRSA (89% MWs, 95% NMWs) and extended-spectrum beta-lactamase-producing *Enterobacteriaceae* (ESBLPE) (83% MWs, 42% NMWs) were increasing in France.

In the univariate analysis (Table 3), variables associated with a KS ≥ 4 were: the category of HCF, male gender, an age between 26 and 35 years, the medical professional status, a shorter current job tenure, working in an ICU and having been trained on AMR control measures. In the multivariate logistic regression analysis, five variables remained positively associated with greater knowledge: working in a UH (adjusted odds ratio, 1.41; 95% CI, 1.16–1.70; *p* < 0.005); age classes 26–35 years (1.43, 1.23–1.67, *p* < 0.0001) and 36–45 years (1.19, 1.01–1.40, *p* = 0.037); medical professional status (3.70, 3.09–4.44, *p* < 0.0001), working in an ICU (1.28, 1.06–1.55, *p* = 0.011) and having been trained on control of AMR within the previous 3 years (1.31, 1.16–1.48, *p* < 0.0001). Working in rehabilitation and long-term care units (0.81, 0.68–0.96, *p* = 0.014) was negatively associated with a higher KS.

**Table 3:** Factors associated with greater knowledge of antimicrobial resistance and infection control measures

Population-based variables	Population (n, %)		Univariate analysis (OR (95% CI))	Multivariate analysis (adjusted OR (95% CI))
	KS < 4	KS ≥ 4		
Type of HCF				
- University hospitals / Cancer centres	1385 (34)	2630 (66)	2.04 (1.83–2.28)	1.41 (1.16–1.70)
- Non-university hospitals	978 (45)	1209 (55)	1.33 (1.18–1.51)	0.99 (0.83–1.19)
- Small, rehabilitation, nursing hospitals	977 (52)	908 (48)	1.00	1.00
- Private clinics	259 (41)	370 (59)	1.54 (1.28–1.84)	1.08 (0.84–1.39)
Gender				
- Male	477 (32)	1022 (68)	1.63 (1.44–1.83)	1.13 (0.97–1.29)
- Female	3064 (43)	4039 (57)	1.00	1.00
Age (years)				
- < 25	511 (41)	726 (59)	1.00	1.00
- 26–35	853 (34)	1634 (66)	1.35 (1.17–1.55)	1.43 (1.23–1.67)
- 36–45	747 (41)	1066 (59)	1.00 (0.87–1.16)	1.20 (1.02–1.41)
- 46–55	764 (45)	937 (55)	0.86 (0.74–1.00)	1.11 (0.94–1.31)
- > 55	291 (48)	321 (52)	0.78 (0.64–0.94)	0.80 (0.64–1.00)
Professional status				
- Medical	218 (17)	1073 (83)	4.12 (3.53–4.79)	3.70 (3.09–4.44)
- Non-medical	3381 (46)	4044 (54)	1.0	1.0
Job tenure (years)				
- < 3	469 (36)	831 (64)	1.00	NA
- 3–10	1070 (40)	1628 (60)	0.86 (0.7–1.0)	
- > 10	1428 (41)	2026 (59)	0.80 (0.7–0.9)	
Working unit				
- Medicine	1015 (37)	1727 (63)	1.00	1.00
- Surgery	617 (38)	993 (62)	0.95 (0.83–1.07)	0.99 (0.86–1.15)
- Intensive care unit	227 (30)	549 (71)	1.42 (1.19–1.68)	1.28 (1.06–1.55)
- Rehabilitation, long-term care	1273 (51)	1218 (49)	0.56 (0.50–0.62)	0.81 (0.68–0.96)
- Emergency	138 (35)	257 (65)	1.09 (0.88–1.36)	0.90 (0.70–1.16)
- Gynaecology-Obstetrics	54 (45)	69 (56)	0.75 (0.52–1.08)	0.98 (0.66–1.45)
- Psychiatry	42 (44)	54 (56)	0.76 (0.50–1.13)	0.91 (0.58–1.43)
- Other	77 (49)	81 (51)	0.62 (0.45–0.85)	0.78 (0.55–1.11)
Previous training in hand hygiene (last 3 years)				
- No	1172 (34)	1597 (32)	1.00	1.00
- Yes	2311 (66)	3442 (68)	1.09 (1.00–1.20)	1.11 (0.98–1.24)
Previous training in control of antimicrobial resistance (last 3 years)				
- No	2283 (69)	3130 (64)	1.00	1.00
- Yes	1001 (31)	1786 (36)	1.30 (1.18–1.43)	1.31 (1.16–1.48)

*KS* Knowledge score, *OR* Odds ratio, *CI* Confidence interval, *NA* Not applicable (numerous missing data)

### Knowledge score and perceptions

After adjustment for variables significantly associated with better knowledge (type of HCF, male gender, age, medical professional status, working unit, and having received training sessions), a higher KS was significantly associated with four cognitive factors: perceived susceptibility (2.33, 95% CI, 1.95–2.78, *p* < 0.0001), positive attitude toward hand hygiene (1.98, 1.65–2.37, *p* < 0.0001), self–efficacy (1.22, 1.09–1.38, *p* < 0.001), and motivation (1.42, 1.24–1.62, *p* < 0.0001) (Table 4).

**Table 4:** Behavioural factors associated with greater knowledge of antimicrobial resistance and infection control measures

Population-based variables	Population (n (%))		Univariate analysis (OR (95% CI))	Multivariate analysis (adjusted OR (95% CI))
	KS < 4	KS ≥ 4		
Perceived susceptibility				
- No agreement	503 (14)	528 (6)	1.00	1.0
- Agreement	3096 (86)	4789 (94)	2.37 (2.05–2.75)	2.33 (1.95–2.78)
Perceived knowledge				
- No agreement	1257 (35)	1910 (37)	1.00	1.0
- Agreement	2342 (65)	3207 (63)	0.90 (0.82–0.98)	1.06 (0.95–1.18)
Intention to adhere				
- No agreement	1232 (34)	1809 (35)	1.00	NA
- Agreement	2367 (66)	3308 (65)	0.95 (0.87–1.04)	
Attitude toward hand hygiene				
- No agreement	442 (12)	348 (7)	1.00	1.0
- Agreement	3157 (88)	4769 (93)	1.92 (1.66–2.22)	1.98 (1.65–2.37)
Perceived behavioural norm				
- No agreement	1755 (49)	2701 (53)	1.00	1.0
- Agreement	1844 (51)	2415 (47)	0.85 (0.78–0.93)	0.95 (0.86–1.05)
Perceived subjective norm				
- No agreement	2025 (56)	3066 (60)	1.00	1.0
- Agreement	1574 (44)	2051 (40)	0.86 (0.79–0.94)	0.98 (0.89–1.09)
Self-efficacy				
- No agreement	944 (26)	1226 (24)	1.00	1.0
- Agreement	2655 (74)	3891 (76)	1.13 (1.02–1.24)	1.22 (1.09–1.38)
Motivation				
- No agreement	647 (18)	758 (15)	1.0	1.0
- Agreement	2952 (82)	4359 (85)	1.26 (1.12–1.41)	1.42 (1.24–1.62)

Adjusted odds ratio: adjusted for type of HCF, gender, age, professional status, working unit and training

See Additional file 1: Table S3 for the formulation of the eight questions about perceptions

*KS* Knowledge score, *OR* Odds ratio, *CI* Confidence interval, *NA* Not applicable (numerous missing data)

### Perceptions of the antimicrobial resistance threat

Most participants perceived AMR as a national problem (Additional file 1: Table S2) (98% MWs, 88% NMWs), while fewer (66% MWs, 40% NMWs) viewed AMR as a local problem, with a low impact on their daily practices (65% MWs, 38% NMWs).

## Discussion

To date, this is the first study evaluating the association between knowledge of AMR epidemiology, the associated control measures, and the individual cognitive factors, including both MWs and NMWs from a national representative population of HCFs. The 74% participation rate was unexpectedly high and may be ascribable to the active participation of IPC teams and the direct physical contact of investigators with ward staff. This large panel therefore accurately reflects the situation in France and enabled comparison of the KS in different categories of HCFs and types of healthcare units.

We found poor knowledge of current AMR epidemiology and modest knowledge of best practices in prevention of cross-transmission. Variations were observed across professional categories, highlighting two profiles. Professionals with the highest knowledge profile were young medical doctors, working in an ICU, recently trained and with awareness of and readiness to act against AMR. This profile perceived poor compliance with hand hygiene as a breach in patient safety, with a willingness to comply with hand hygiene recommendations. The 26–35-year age class working in UHs was associated with greater knowledge, possibly reflecting improved and fresh education on the topic during medical or nursing studies. On the other hand, low knowledge was found among nurse aides from small LTCFs. Nurse aides are key people for infection control. They routinely contribute to patient care and diaper changes, with a high risk of hand contamination and subsequent transmission [18]. This strongly suggests that knowledge should primarily be improved in that population. Small HCFs should also be a target for education as they may suffer of a lack of IPC human resources.

Fifteen years after the introduction of AHR in French healthcare settings [17, 19], knowledge of hand hygiene best practices still appeared poor. AHR was considered less effective than antiseptic or plain soap in a significant proportion of respondents, as high as 50% of NMWs, which was very disappointing given the multiple national campaigns promoting AHR and the use of AHR consumption as a national quality indicator. Two previous studies reported that medical students considered poor hand hygiene compliance as one of the least important contributors to AMR [11, 20]. In consequence, educational messages provided by IPC teams should be simplified, focused on the reasons for and consequences of poor hand hygiene practices and be tailored to the healthcare professionals involved.

Furthermore, less than 50% of HCWs thought that hand hygiene was more important after than before a contact with patients. These results illustrate a general misconception of hand hygiene best practices, even though reported consumption of AHR in France is fairly high compared to other European countries [21]. Healthcare-associated infections are the result of a complex chain, including the many individuals involved in patient care. The consequences of poor hand hygiene compliance are intangible for front-line staff, not considering the actual burden of AMR for patients as a consequence of their individual practices. The perception of AMR as a national problem but not a local or individual one supports this hypothesis. Accurate feedback of local data may improve awareness of HCWs [22].

.HCWs still believe they need to wear gloves for contact precautions despite its withdrawal from French recommendations in 2010. Several guidelines have recently been issued for the control of MDRO transmission, with evolving recommendations (e.g. the debated need for contact precaution for ESBLP-*E. coli*) [1, 23]. These recurrent changes in recommendations may be confusing for HCWs, complicating the implementation of good practices. Sixty-eight percent of HCWs reported having received training on hand hygiene during the last 3 years. This proportion, albeit high, may be considered insufficient. Education and training of HCWs are one pillar of infection control programmes and efforts must be made to implement regular courses and target all HCW categories [24]. However, formal training should be included in a larger programme including combined measures, according to the rules of bundling and multifaceted interventions: reminders at the workplace, audit and feedback, use of AHR consumption as a performance indicator, leadership, incentive and rewards … [25]. For example, AHR consumption is a publicly released quality indicator for all healthcare facilities in France, and facilities are urged to use AHR consumption as an internal quality indicator; most healthcare facilities are registered to take part in national hand hygiene day, on the 5th of May, as well as in the national yearly week of patient safety. Until now, educational programmes have usually been based on classic presentations with lectures given to a passive audience. New technologies such as simulation, virtual reality, serious games and e-learning applications, playing with the trainee’s emotions, bring new possibilities to the field of medical training and could lead to valuable improvement in learning outcomes [26]. After adjustment for confounding variables, a higher KS was significantly associated with four cognitive factors: perceived susceptibility, attitude toward hand hygiene, self-efficacy, and motivation. Our survey, as previously described [27], suggests a perceived lack in patient safety by HCWs when hand hygiene in inadequately performed. One may consider that the perceived susceptibility, i.e. the perceived risk to patient, which was the strongest factor linked to higher knowledge, derived from the higher knowledge by itself. Nevertheless, it is unknown whether the other cognitive factors impact higher knowledge, or whether higher knowledge obtained from other sources, such as training sessions or medical education, translates into more belief in and perception of the importance of hand hygiene. The interactions probably are intricate, suggesting that training on hand hygiene and AMR is critical in shaping beliefs in and perceptions of the control of AMR. A new approach based on psychologically tailored hand hygiene interventions regarding MDRO has recently been described [28]. Tailored intervention based on the Health Action Process Approach (HAPA) led to better compliance with hand hygiene, with in turn a decrease in the MDRO infection rate.

Understanding the impact of individual infection control behaviours on AMR spread may increase the likelihood of compliance. An adapted approach is needed to heighten an individual’s understanding. A unique strategy is not sufficient in a such context and efforts should be made to implement personalised and multiple tools. One approach could be supported by evidence-based medicine. A recent study stated that recommendations appear to be imposed on medical students and junior physicians without reference to the scientific evidence, which therefore does not encourage high compliance with hand hygiene [22]. Feedback of local data could increase awareness among HCWs, while demonstrating threats in their own setting and the consequences of their own practices. On the other hand, social norms (perceived behavioural and subjective norms) are independent of awareness, but surveys have demonstrated that they shape hygiene behaviours [17, 29]. For instance, perceived peer handwashing frequency significantly impacted the behaviour of professionals. Intervention regarding social norms could be a complementary approach.

Our survey had some limitations. Firstly, the study was performed in France and was probably not representative of the healthcare systems of other countries. Indeed, to our knowledge, only one study has been conducted in several European countries, but focused on antibiotic prescribing and AMR among medical students [11]. Secondly, the questionnaire was unique and questions could have been understood differently by individuals according to their professional status. Hence, use of a 7-point scale permitted a large range of responses and more precision [30]. Thirdly, it is likely that the respondents were more motivated and better informed than non-respondents, thus increasing the rate of positive responses. However, the high participation rate could offset this bias. Finally, some answers may have been collective rather than individual, thereby falsely increasing KS.

## Conclusions

In view of insufficient knowledge among HCWs, training should be extended to all HCW categories, and simplified to address simple control measures. New strategies to enhance awareness should probably incorporate different professional beliefs and contextual institutional factors, suggesting new possible areas of intervention. Non-medical HCWs with a lower educational level and small HCFs should be prioritised, by adapting IPC tools and education methods used in large university hospitals. Designing new strategies for the effective implementation of evidence-based infection control practices is essential and should be a priority at all levels.

## Supplementary information

**Additional file 1: Table S1.** Knowledge of antimicrobial resistance and infection control measures. **Table S2.** Perceptions regarding antimicrobial resistance and control measures. **Table S3.** Questions on perception of antimicrobial resistance and control measures.

## Data Availability

Tools (knowledge and perception questionnaires) are included in this published article and its supplementary information files. The datasets generated and/or analysed during the current study are not publicly available.

## Abbreviations

AHR: Alcohol-based hand rub
AMR: Antimicrobial resistance
ESBLP: Extended-spectrum beta-lactamase-producing
HAPA: Health Action Process Approach
HCF: Healthcare facility
HCW: Healthcare worker
ICU: Intensive care unit
IPC: Infection prevention and control
KS: Knowledge score
LTCF: Long-term care facilities
MDRO: Multidrug-resistant organisms
MRSA: Methicillin-resistant *Staphylococcus aureus*
MW: Medical healthcare worker
NMW: Non-medical healthcare worker
NUPH: Non-university public hospitals
Q1: 25th percentile
Q3: 75th percentile
SD: Standard deviation
UH: University hospitals or referral centres for cancer
