# Supplementary material for: Awareness among French healthcare workers of the transmission of multidrug resistant organisms: a large cross-sectional survey
Source: Antimicrob Resist Infect Control. 2019 Nov 12;8:173. doi: 10.1186/s13756-019-0625-0 (PMC6852912; doi:10.1186/s13756-019-0625-0)
Supplement: Supplementary file 1 — Additional file 1: Table S1. Knowledge of antimicrobial resistance and infection control measures. Table S2. Perceptions regarding antimicrobial resistance and control measures. Table S3. Questions on perception of antimicrobial resistance and control measures. [file 13756_2019_625_MOESM1_ESM.docx]

**Table S1 - Knowledge of antimicrobial resistance and infection control measures**

|  | **Total**  **(58 HCFs)** | | **University hospitals**  **centres /**  **Cancer centres**  **(n= 9)** | | **Non-university hospitals**  **(n= 10)** | | **Small hospitals, rehabilitation centres, nursing homes (n= 29)** | | **Private clinics**  **(n= 10)** | |
| --- | --- | --- | --- | --- | --- | --- | --- | --- | --- | --- |
|  | **MW**  **(n (%))** | **NMW**  **(n (%))** | **MW**  **(n (%))** | **NMW**  **(n (%))** | **MW**  **(n (%))** | **NMW**  **(n (%))** | **MW**  **(n (%))** | **NMW**  **(n (%))** | **MW**  **(n (%))** | **NMW**  **(n (%))** |
| **Indication for hand hygiene (correct answer)**  After contact with the patient’s environment? (yes)  When performing hand hygiene? (before contact)  Best product for hand hygiene? (alcoholic handrub) | 1102 (88)  536 (42)  955 (76) | 5694 (80)  3491 (48)  3527 (48) | 1097 (85)  533 (41)  952 (74) | 5699 (77)  3495 (47)  3531 (47) | 245 (86)  113 (40)  215 (76) | 1443 (76)  869 (46)  800 (42) | 73 (76)  28 (29)  66 (69) | 1308 (73)  822 (46)  771 (43) | 73 (82)  44 (49)  60 (67) | 422 (78)  267 (49)  268 (50) |
| **Indication for glove use (correct answer)**  Wearing gloves for contact with intact skin, standard precautions? (no)  Wearing gloves for contact with intact skin, contact precautions? (no) | 992 (84)  119 (9) | 5389 (81)  409 (6) | 988 (77)  118 (9) | 5394 (73)  410 (5) | 211 (74)  25 (9) | 1395 (73)  74 (4) | 70 (73)  21 (22) | 1232 (69)  107 (6) | 58 (65)  8 (9) | 383 (71)  23 (4) |
| **Knowledge of epidemiology of MDROs (correct answer)**  Decreasing methicillin-resistant *Staphylococcus aureus* rates? (yes)  Increasing extended-spectrum beta-lactamase-producing *Enterobacteriaceae* rates? (yes)  Transmission of methicillin-resistant *Staphylococcus aureus*? (hands) | 147 (12)  1068 (84)  1098 (87) | 363 (5)  3139 (43)  4944 (70) | 147 (11)  1065 (83)  1094 (85) | 363 (5)  3143 (42)  4949 (67) | 25 (9)  219 (77)  231 (81) | 81 (4)  757 (34)  1295 (68) | 13 (13)  81 (84)  83 (86) | 116 (6)  647 (36)  1055 (59) | 5 (5.6)  66 (74.2)  71 (79.8) | 43 (8)  222 (41)  356 (66) |

Abbreviations: HCFs, healthcare facilities; MWs, medical healthcare workers; NMWs, non-medical healthcare workers; MDROs, multidrug-resistant organisms

Since not all questions were answered, the denominator may vary across questions.

**Table S2 - Perceptions regarding antimicrobial resistance and control measures**

| **Perception-based variables** | **Total**  **(58 HCFs)** | | **University hospitals / cancer centres (n= 9)** | | **Non-university hospitals  (n= 10)** | | **Small hospitals, rehabilitation centres, nursing homes (n= 29)** | | **Private clinic  (n= 10)** | |
| --- | --- | --- | --- | --- | --- | --- | --- | --- | --- | --- |
|  | **MW**  **(n (%))** | **NMW**  **(n (%))** | **MW**  **(n (%))** | **NMW**  **(n (%))** | **MW**  **(n (%))** | **NMW**  **(n (%))** | **MW**  **(n (%))** | **NMW**  **(n (%))** | **MW**  **(n (%))** | **NMW**  **(n (%))** |
| **Health belief perception** |  |  |  |  |  |  |  |  |  |  |
| Perceived susceptibility | 1170 (91) | 6715 (90) | 737 (90) | 2947 (92) | 260 (91) | 1697 (89) | 92 (93) | 1576 (88) | 81 (91) | 495 (92) |
| Perceived knowledge | 553 (43) | 4996 (67) | 305 (37) | 1993 (62) | 135 (47) | 1285 (68) | 68 (69) | 1309 (73) | 45 (51) | 409 (76) |
| Intention to adhere/perceived practice | 582 (45) | 5093 (69) | 348 (42) | 2166 (68) | 137 (48) | 1302 (68) | 57 (58) | 1235 (69) | 40 (45) | 390 (72) |
| Attitude toward hand hygiene | 1134 (88) | 6792 (91) | 718 (88) | 2945 (92) | 244 (86) | 1728 (91) | 93 (94) | 1621 (91) | 79 (89) | 498 (92) |
| Perceived behavioural norm | 446 (34) | 3814 (51) | 263 (32) | 1596 (50) | 109 (38) | 950 (50) | 37 (37) | 954 (53) | 37 (42) | 314 (58) |
| Perceived subjective norm | 329 (25) | 3296 (44) | 162 (20) | 1350 (42) | 94 (33) | 806 (42) | 39 (39) | 871 (49) | 34 (38) | 269 (50) |
| Self-efficacy | 847 (66) | 5699 (77) | 511 (62) | 2415 (75) | 189 (66) | 1445 (76) | 84 (84) | 1395 (78) | 63 (71) | 444 (82) |
| Motivation | 994 (77) | 6317 (85) | 614 (75) | 2732 (85) | 222 (78) | 1600 (84) | 91 (92) | 1518 (85) | 67 (75) | 467 (86) |
| **Perceived threat of antimicrobial resistance** |  |  |  |  |  |  |  |  |  |  |
| National level | 1247 (98) | 6491 (88) | 791 (98) | 1669 (89) | 276 (98) | 1520 (86) | 96 (97) | 2846 (90) | 84 (94) | 456 (86) |
| Local level | 837 (66) | 2909 (40) | 590 (73) | 734 (39) | 170 (61) | 497 (28) | 57 (58) | 1537 (49) | 20 (22) | 141 (27) |
| Daily practice | 835 (65) | 2755 (38) | 552 (68) | 713 (38) | 181 (65) | 554 (31) | 69 (70) | 1318 (42) | 33 (37) | 170 (32) |

Footnote

HCFs. Health care facilities; MWs. Medical healthcare workers; NMWs. Non-medical healthcare workers

**Table S3 - Questions on perception of antimicrobial resistance and control measures**

| **Perception category** | **Question** |
| --- | --- |
| Perceived susceptibility | “Non-compliance with hand hygiene entails risk to the patient” |
| Perceived knowledge | “I know the recommended indications for hand hygiene” |
| Intention to adhere/perceived practice | “I do perform hand hygiene as recommended” |
| Attitude toward hand hygiene | “I think that hand hygiene is a useful measure” |
| Perceived behavioural norm | “My colleagues perform hand hygiene according to the recommended guidelines” |
| Perceived subjective norm | “My hand hygiene is taken as an example by my colleagues” |
| Self-efficacy | “I can comply with hand hygiene according to recommended guidelines” |
| Motivation | “I can improve my compliance with hand hygiene” |
